# Evaluation of Health Care System Reform in Hubei Province, China

**DOI:** 10.3390/ijerph110202262

**Published:** 2014-02-21

**Authors:** Shuping Sang, Zhenkun Wang, Chuanhua Yu

**Affiliations:** 1 School of Public Health, Wuhan University, 115 Donghu Road, Wuhan 430071, China; E-Mails: teaeggnew@163.com (S.S.); wongzhenkun@gmail.com (Z.W.); 2 Global Health Institute, Wuhan University, 115 Donghu Road, Wuhan 430071, China

**Keywords:** health care system reform, evaluation, indicators, China

## Abstract

This study established a set of indicators for and evaluated the effects of health care system reform in Hubei Province (China) from 2009 to 2011 with the purpose of providing guidance to policy-makers regarding health care system reform. The resulting indicators are based on the “Result Chain” logic model and include the following four domains: Inputs and Processes, Outputs, Outcomes and Impact. Health care system reform was evaluated using the weighted TOPSIS and weighted Rank Sum Ratio methods. Ultimately, the study established a set of indicators including four grade-1 indicators, 16 grade-2 indicators and 76 grade-3 indicators. The effects of the reforms increased year by year from 2009 to 2011 in Hubei Province. The health status of urban and rural populations and the accessibility, equity and quality of health services in Hubei Province were improved after the reforms. This sub-national case can be considered an example of a useful approach to the evaluation of the effects of health care system reform, one that could potentially be applied in other provinces or nationally.

## 1. Introduction

In April 2009, China had announced a health reform blueprint for achieving universal coverage by 2020 [1]. The plan focuses on major structural change in five areas over the next three years: (a) Expanding the medical security system, (b) establishing the essential medicines system, (c) strengthening the capacity of primary care facilities, (d) reducing gaps in coverage of public health services, and (e) reforming the organization and financing mechanisms for public hospitals on a pilot basis. The 2009–2011 implementation plan is backed by an investment of 850 billion Yuan (124 billion USD) from the central and regional governments [2]. To meet the overall design and working requirements for expanding health care system reform in China and to ensure that health care system reform will proceed in a planned and orderly manner, the Center for Health Statistics and Information (CHIS) has conducted an evaluation titled *Monitoring and Evaluation of China’s Health Care System Reform Project* [3]. Hubei Province and Shanghai Municipality were chosen as the first pilot areas for the research. The establishment of a scientific evidence-based monitoring and evaluation system is needed to answer a series of questions: How has Hubei Province’s health care system performed? Will China’s health care system reform efforts improve the health system sub-nationally? In what particular areas has Hubei performed well or poorly? Therefore, the health care system reform evaluation has three aims: managing the health investment, monitoring the reform process and providing evidence-based assessment of reform policies. In this study, we conducted a comprehensive evaluation of health care system reform in Hubei Province from 2009 to 2011 by establishing an indicator system for performance evaluation. Our assessment was then translated into a set of suggestions for health care system reform in Hubei Province.

Three evaluation frameworks for health care system reform have been described [4]. The first is the “Control Knobs” method proposed by the World Bank and Harvard University in 2004, the second is the “Building Blocks” model proposed by WHO in 2006 [5], and the third is the “Primary Health Care Evaluation Framework”. Many countries use their evaluation frameworks to monitor and evaluate health care system reform. Britain, for example, proposed the NHS Performance Assessment Framework in 1999 [6]. The subject of the framework is an indicator system of the National Health System’s performance, which includes health service quality, efficiency and outcome. The framework highlights six domains, including health improvement, accessibility of health equity, effective utilization of the health service, efficiency, patient experience and health outcomes. In Mexico, the aim of the evaluation is to measure effective coverage [7,8]. Australia proposed a continuous evaluation framework, which mainly targets hospital performance. Indicators of the framework include medical quality, health outcomes, and clinical indicators, among others [9]. The United States’ health policy experts proposed benchmarks to evaluate whether a country’s health system reform is successful [10,11]. The central premise is that disease and disability reduce the opportunities available to individuals and that the principle of equal opportunity provides a basis for regulating a health care system. The same theory can be extended to look beyond the point of delivery of health care to the social determinants of health.

Because health care system reform in China has its own characteristics, China’s Center for Health Statistics and Information (CHIS) invited experts from the WHO to design an evaluation framework for China. This evaluation framework refers to the international experience and the “Results Chain”. It provides a set of indicators, which focus on the evaluation of reform for 2009–2011. In addition, this system is proposed based on the practicalities of health care system reform in China. However, it is difficult to use the same indicator system in different provinces because China is such a large and heterogeneous nation. Therefore, we selected the indicators from this system and added other indicators in consideration of the local health development level in Hubei Province. We then weighted each indicator using scientific methods and comprehensively evaluated health care system reform in Hubei Province from 2009 to 2011.

## 2. Methods

### 2.1. Data Source

The data used in this study are derived from *Monitoring Progress Table on Recent Focus Work of the Health Care System Reform* (*i.e.*, Health care system reform Monitoring Table), *Health Statistics Yearbook of Hubei Province* [12,13,14], and statistical information from departments related to health care system reform in Hubei Province from 2009 to 2011.

### 2.2. Establishment of Indicator System

**Figure 1:**
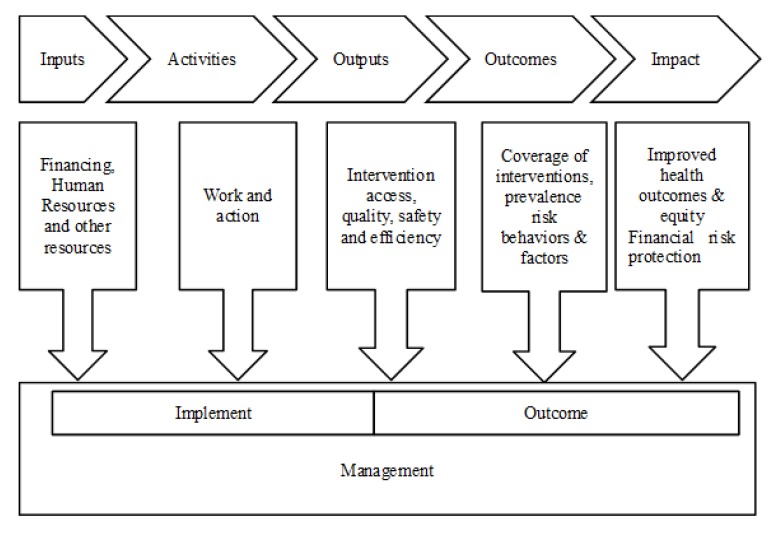
Logical frame of Results Chain.

The foundation of our study is *Monitoring and* *Evaluation* *Framework of China’s Health Care System Reform,* which was proposed by the WHO and is based on the logical framework of a “Results Chain”. The “Results Chain” is results-oriented, with evaluation as a component of promoting intervention, strategy and policy (Figure 1). 

As we know, what all the evaluation frameworks described for health care system reform have in common is consensus that monitoring and evaluation must address performance in terms of both health system measures—availability, access, quality, efficiency, and population health measures—health status, responsiveness, user satisfaction, financial risk protection. Therefore, after referring to the international experience and the “Results Chain”, we developed the evaluation indicator system for Hubei Province based on four domains: Input and Process, Output, Outcome, and Impact. We screened the indicators based on importance, briefness, sensibility, availability and comparability.

### 2.3. Determination of the Weights

Satty’s Weighting Method of Analytic Hierarchical Process was used to determine the weights of indicators at different levels in the indicator system [15]. The team establishing the weights was composed of 13 experts in the field of health reform and performance evaluation representing different universities, institutes and health departments such as the Health Care Center, Centers for Disease Control (CDC) and Health Information Statistics Center. The importance of the indicators was scored by the experts, assessment targets were resolved, and evaluation indicators at different levels were then obtained [16]. Four major steps were used to compute the weights:

1. The sum of each column is calculated using the pair-wise comparison matrix. In our study, we use “The 9-point scale for comparative judgments” suggested by Satty(1980) to transform the verbal judgments into numerical quantities [17]. 

2. The initial weights are calculated by Equation (1):

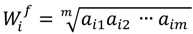
(1)
where *W'_j_* is the *j*th indicator’s initial weight, *j* = 1,2∙∙∙*m*, *m* represents the number of the indicators, and *a_j1_*, *a_j2_*∙∙∙*a_jm_* represents the elements of the *j*th line in the judgment matrix.

3. Equation (2) is used to calculate the normalized weight coefficient to obtain the weight of each indicator:

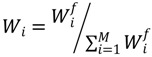
(2)

4. Paired comparison consistency is assessed to test the logic of the weights. The consistency ratio (*CR*) is determined by Equation (3):

*CR* = *λ_max_*/*RI(m-1)* (3)

where *λ_max_* is the maximum eigenvalue, m represents the number of the indicators, and RI is a random index; when m is equal to 3 and 8, RI is 0.58 and 1.41, respectively. The Consistency Ratio should be less than 0.1; otherwise, the decision should be revised [18]. 

The final weight of each indicator is calculated by multiplying the weights of the level 1, 2 and 3 evaluation indicators. 

### 2.4. Synthesized Evaluation Methods

The TOPSIS method and Weighted Rank-Sum Ratio (WRSR) were used to evaluate health care system reform performance in this study. The TOPSIS method eliminates the influence of different indicator dimensions by using the same trending method and the normalization method to process the original data from the evaluation indicators to determine the best and worst solutions in finite schemes in the data matrix based on normalization. The relative similar degree *C_i_* between the evaluation object and the best solution is calculated by the Equation (4):

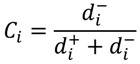
(4)
where *i* = 1,2…*n*, *n* is the number of the evaluation objects, *d_i_*^+^ is the distance between an evaluation object’s value and the positive ideal solution, and *d_i_*^-^ is the distance between an evaluation object’s value and the negative ideal solution. A larger *C_i_* value indicates better comprehensive benefits and higher evaluation [19]. 

The basic idea of the Weighted Rank Sum Ratio (WRSR) method is to convert the original data matrix by rank transformation and then to calculate the ratio of each appraisal object rank to its ideal maximum rank by the Equation (5):

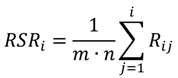
(5)
where *R_ij_* is the rank of the element in the *i*th line of the *j*th column, *i* = 1,2∙∙∙*n*, *j* = 1,2∙∙∙*m*. Equation (5) is used to perform the weighted calculation to perform the Weighted Rank Sum Ratio (WRSR), where *W_j_* is the weight of the *j*th indicator:

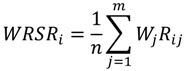
(6)

A larger *WRSR_i_* value indicates better comprehensive benefits and performance [20]. 

### 2.5. Sensitivity Analysis of the Weights

If *W'_j_* is the *j*th indicator’s initial weight, 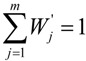, then we can calculate the marginal weight coefficient [21] *W'_r_* and *W'_s_* by using the Equations (7) and (8):

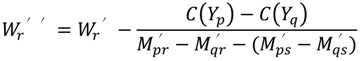
(7)

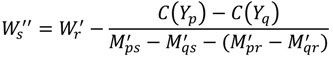
(8)

## 3. Results

### 3.1. Screening Results of Evaluation Indicator

The indicator system of the health care system reform evaluation is composed of four parts: Inputs and Process, Outputs, Outcomes, and Impact. The final evaluation indicator system and weights are shown in Table 1, Table 2, Table 3 and Table 4. There are 20 indicators in the “Input and Process” domain, which mainly reflects conditions of the policy and management of health care system reform, health financing, infrastructure, health human resources, service system, and information of health care system reform. There are 30 indicators in the “Outputs” domain, which mainly reflect conditions of accessibility, quality, safety and efficiency of interventions. There are nine indicators in the “Outcomes” domain, which mainly reflect coverage of interventions and changes in people’s health behavior. Finally there are 12 indicators in the “Impact” domain, which mainly reflect the long-term effects and influences of health care system reform activities.

### 3.2. The Evaluation Results of the Reform

Table 5 presents the evaluation results. From 2009 to 2011, performance in three of the domains (Inputs and Process, Outputs, Impact) of health care system reform increased year by year in both evaluation methods, whereas the performance ranking results in the “Outcomes” domain differed across the different methods. In general, the total performance of the health care system reform in Hubei Province increased year by year from 2009 to 2011 (Table 6).

### 3.3. Sensitivity Analysis of the Weights

Table 7 shows the marginal weight coefficient of *M*_1_, the first indicator of the system. Every marginal weight coefficient is larger than 1, which means the marginal weight is not in the allowable range. We then calculated the marginal weight coefficient of the other indicators, and no marginal weight coefficient fell within the allowable range. Therefore, we can say that the weight of each indicator is insensitive [22].

**Table 1:** Indicator system for healthcare.

Grade-1 Indicators (Weight)	Grade-2 Indicators (Weight)	Grade-3 Indicators (Weight)	Synthetic Weight
Input and process (0.2)	Health resource (0.30)	Total health expenditure(TEH) (0.22)	0.0132
		% of TEH in GDP (0.14)	0.0084
		Total medical institutes (0.08)	0.0048
		Medical institutes per thousand people (0.10)	0.0060
		Total medical beds(0.07)	0.0042
		Medical beds per thousand people (0.09)	0.0054
		Total health personnel (0.08)	0.0048
		Health personnel per thousand people (0.09)	0.0054
		Certified doctor per thousand people (0.07)	0.0042
		Registered nurse per thousand people (0.06)	0.0036
	Total investment in reform (0.15)	Government special grants (1.00)	0.0300
	Basic medical insurance (0.15)	Government special grants (0.50)	0.0150
		government subsidies for basic medical insurance (0.50)	0.0150
	Essential medicines (0.10)	Government special grants (0.50)	0.0100
		Coverage of essential medicines (0.50)	0.0100
	Basic medical health service system (0.10)	Government special grants (0.50)	0.0100
		Training basic level sanitary personnel (0.50)	0.0100
	Basic public health services (0.10)	Government special grants (0.50)	0.0100
		*per capital* public health expenditures (0.50)	0.0100
	Public hospital reform (0.10)	Government special grants (1.00)	0.0200
Output (0.35)	Improve health service accessibility (0.35)	The average outpatient expenditure(0.18)	0.0221
		The average hospitalizing expenditure (0.18)	0.0221
		% of average medicine fee in outpatient expenditure (0.19)	0.0233
		% of average medicine fee in inpatient expenditure (0.19)	0.0233

**Table 2:** Indicator system for healthcare (continued).

Grade-1 Indicators (Weight)	Grade-2 Indicators (Weight)	Grade-3 Indicators (Weight)	Synthetic Weight
Output (0.35)	Improve health service accessibility (0.35)	Number of village clinics (0.08)	0.0098
		Number of township health centers (0.09)	0.0110
		Number of community health service centers(stations) (0.09)	0.0110
	Improve the equity of the health service (0.20)	% of government health expenditures in THE (0.07)	0.0050
		% of social health expenditures in THE (0.07)	0.0050
		% of out-of-pocket payments in THE (0.07)	0.0050
		Number of hospital outpatient visits (0.05)	0.0036
		Number of Basic medical institutions outpatient visits (0.05)	0.0036
		Number of village clinics outpatient visits (0.05)	0.0036
		Number of professional public health agency outpatient visits (0.05)	0.0036
		Rate of establishing individual health profiles in urban residents (0.06)	0.0040
		Rate of establishing individual health profiles in rural residents (0.06)	0.0040
		Number of patients with hypertension management (0.07)	0.0046
		Number of patients with diabetes mellitus (0.07)	0.0046
		Number of individuals aged< 15 years is a catch-up program for hepatitis B vaccinations (0.07)	0.0046
		Number of rural women undergoing cervical cancer screening (0.06)	0.0043
		Number of rural women undergoing breast cancer screening (0.06)	0.0043
		Number of rural women of childbearing age who take folate daily (0.06)	0.0043
		Number of impoverished patients who accept free cataract surgeries (0.05)	0.0032
		Coverage of health toilet (0.04)	0.0027

**Table 3:** Indicator system for healthcare (continued).

Grade-1 Indicators (Weight)	Grade-2 Indicators (Weight)	Grade-3 Indicators (Weight)	Synthetic Weight
Output (0.35)	Improve the quality of the health service (0.25)	Consistent rate of diagnoses between patients and inpatients (0.19)	0.0166
		Consist rate of diagnosis before and after operation (0.19)	0.0166
		Consistent rate of agreement between clinical and pathological diagnoses (0.19)	0.0166
		Rate of correct diagnosis within 3 days (0.19)	0.0166
		Nosocomial infection rates (0.24)	0.0210
	Increase the efficiency of the health service (0.20)	Total number of outpatient visits (0.15)	0.0105
		Total number of inpatients (0.15)	0.0105
		Average number of patients per day per doctor (0.18)	0.0126
		Average inpatients per day per doctor (0.18)	0.0126
		Average length of stay (0.18)	0.0126
		Bed occupancy rate (0.16)	0.0112
Outcome (0.25)	Coverage of intervention (0.60)	Rate of prenatal care (0.16)	0.0240
		Rate of postnatal care (0.16)	0.0240
		Rate of hospital delivery (0.17)	0.0255
		Systematic management rate for pregnant women (0.17)	0.0255
		Rate of systematic management of children <3 years of age (0.17)	0.0255
		Rate of systematic management of children <7 yeas of age (0.17)management rate (0.17)	0.0255
	Risk factors (0.40)	Incidence of pulmonary tuberculosis (0.33)	0.0330
		Incidence of viral hepatitis (0.31)	0.0310
		Incidence of AIDS (0.36)	0.0360
Impact (0.20)	Health status (0.40)	Infant mortality rate (0.26)	0.0208
		Mortality rate of children under 5 (0.26)	0.0208
		Maternal mortality rate (0.26)	0.0208
		Life expectancy (0.22)	0.0176

**Table 4:** Indicator system for health care (continued).

Grade-1 Indicators (Weight)	Grade-2 Indicators (Weight)	Grade-3 Indicators (Weight)	Synthetic Weight
Impact (0.20)	Economic risk- sharing (0.30)	Number of urban employees with basic health insurance (0.11)	0.0063
		Coverage of urban employees with basic health insurance (0.23)	0.0135
		Number of urban residents with basic health insurance (0.11)	0.0064
		Coverage of urban employees with basic health insurance (0.23)	0.0136
		Number of new rural cooperative medical systems (NCMS) (0.11)	0.0065
		Coverage of new rural cooperative medical systems (0.23)	0.0137
	Social satisfaction (0.30)	Outpatient service satisfaction (0.50)	0.0300
		Inpatient service satisfaction (0.50)	0.0300

**Table 5:** Rankings for performanceevaluation of healthcare reform in Hubei Province 2009–2011.

Year	Input and Process				Output				Outcome				Impact			
	TOPSIS		WRSR		TOPSIS		WRSR		TOPSIS		WRSR		TOPSIS		WRSR	
	*C_i_*	Rank	*WRSR*	Rank	*C_i_*	Rank	*WRSR*	Rank	*C_i_*	Rank	*WRSR*	Rank	*C_i_*	Rank	*WRSR*	Rank
2009	0.573	3	0.538	3	0.327	3	0.352	3	0.388	3	0.453	3	0.609	3	0.537	3
2010	0.724	2	0.584	2	0.359	2	0.379	2	0.568	1	0.562	1	0.617	2	0.590	2
2011	0.786	1	0.669	1	0.361	1	0.401	1	0.543	2	0.508	2	0.676	1	0.528	1

**Table 6:** Rankings for comprehensive performanceevaluation of healthcare reform in Hubei Province 2009–2011.

Year	TOPSIS		WRSR	
	*C_i_*	Rank	*WRSR*	Rank
2009	0.658	3	0.623	3
2010	0.737	2	0.658	2
2011	0.781	1	0.689	1

**Table 7:** Marginal weight coefficients of *M*_1_.

Indicators	Marginal weight coefficient
*M*_1_, *M*_2_	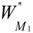 =2.250, 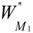 = 3.717
*M*_1_, *M*_3_	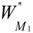 =2.232, 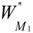 = 2.392
*M*_1_, *M*_4_	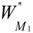 =2.258, 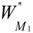 =3.251
*M*_1_, *M*_5_	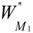 =1.297, 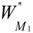 =3.291
*M*_1_, *M*_6_	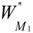 =1.084, 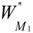 =5.345
*M*_1_, *M*_7_	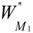 =2.141, 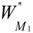 =2.518
*M*_1_, *M*_8_	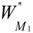 =2.172, 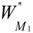 =4.490
*M*_1_, *M*_9_	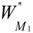 =2.230, 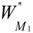 =3.987

## 4. Discussion

### 4.1. Evaluation Methods and Evaluation Results

This study utilized two comprehensive evaluation methods. While the TOPSIS method is more precise, the Weighted Rank-Sum Ratio method can be used as a comprehensive index specialized for statistical analysis that does not introduce subjective variables; thus, it avoids the deficits of the subjective weighting method and demonstrated strong comprehensive ability [23]. Relatively stable evaluation results can be obtained when these two methods are used together. 

Based on our results, we conclude that performance in the Input and Process, Output and Impact domains increased yearly from 2009 to 2011 in Hubei Province. The Input and Process domain was more efficient than the others, and the values of *Ci* and WRSR were the highest. This finding indicates that health care system reform led to better results with regard to health input and health resource allocation. However, increasing performance was not observed in the Output domain, and the values of *Ci* and WRSR were lower. The Output domain covers improvements in accessibility, equity, quality and efficiency of health care services. In this study, we used indicators such as Average Number of Outpatients/Inpatient Expenditures, Percentage of Revenues Allocated to Outpatient/Inpatient Medications to measure accessibility. These indicators were improved and accessibility was enhanced in 2011. However, health expenditures were still high and the level of health accessibility in Hubei Province was still lower than the world average. Additionally, the composition of Total Health Expenditure (THE) was unreasonable, as the proportion of Out-of-Pocket Payment was still higher than government (or social) health expenditures. This disparity adversely affected Output performance. It is worth noting that the performance in the Outcome domain increased and reached its peak in 2010 and decreased during the next year (2011), possibly because the incidences of some communicable diseases (e.g., pulmonary tuberculosis and AIDS) were increasing in 2011, which required more work in communicable disease control. In general, the performance of health care system reform in Hubei Province increased from 2009 to 2011, and health care system reform has played an active role in the health care system in Hubei Province.

### 4.2. Characteristics and Challenges of Evaluation of a Health Care System Reform Program in China

In the past, China lacked macroeconomic monitoring and evaluation of the basic health care system and the effects of the health system. Since the 1970s, most of the influential assessment reports of the Chinese health system have been completed by international organizations, such as the Assessment of China’s Primary Health Care System in the late 1970s by the World Health Organization [24]. The program on health performance evaluation of nations, carried by World Health Organization in 2000, led to widespread concern in China and in-depth reflection on health equity. In 2005, China’s Development Research Center of the State Council conducted a review and evaluation of health care system reform, analyzing the effectiveness of and problems with health services development and health reform after a new China was founded [25]. However, evaluations of the development of health care system reform are still not very mature. Health care system reform involves complicated dynamics, and there are regional differences; thus, systematic and comprehensive evaluation of health care system reform work faces many challenges, including: (1) Evaluation of health reform in China is still in the early stages of development, so the main body of information is small; therefore, more attention must be paid to measuring the performance of health care system reform; (2) Regional differences and difficulties in data collection exist. Given China’s unbalanced regional development, there are great differences in economic and social development, natural and geographical conditions, systematic construction of health resources and reform foundations, and it is not viable to use a national unified evaluation system to measure the performance of health care system reform in all regions. (3) The framework of health reform evaluation needs to be constantly improved as the health system continues to develop and undergo reform.

### 4.3. Suggestions for Health Care System Reform in Hubei

The first three years of China’s new health care system reform were 2009 to 2011, a key period for the five priority reform programs. As health care system reform in China differs from that of more developed Western countries, the challenges we face include more technical risks in policy design and more risks in the implementation of management and supervision [26]. Therefore, it is of great importance to establish a health care system reform evaluation system suited to both provincial and national conditions. The health resources and investments in the health care system reform in Hubei Province have increased year by year since the reform began. There remains, however, a certain gap between the optimum value and the actual values calculated using the two evaluation methods, indicating that performance in the Inputs and Process domain could be further improved. 

The Outputs evaluation consisted of four evaluation areas: health service accessibility, equity, quality and efficiency. Most aspects of the health services in Hubei Province have improved since health care system reform. For instance, the accessibility of health services has been improved as medicine fees have decreased; the proportion of government health expenditures increased year by year, and free public health services for special groups improved the equity of health services, among other improvements. However, in terms of health financing equity, the proportions of Hubei provincial government health expenditures, social health expenditures and out-of-pocket payments in THEs (Total Health Expenditures) were 24.62%, 28.66% and 46.72%, respectively, in 2009. These numbers increased to 30.02%, 30.32% and 39.66%, respectively, in 2011. Out-of-pocket payments still account for the largest proportion of THEs. Therefore, out-of-pocket payments need to be further controlled, and the personal burden on residents needs to be reduced, so that the problem of the high costs of medical treatment can truly be resolved. Meanwhile, in terms of enhancing the efficiency of health resources utilization, Integrated Medical could be considered a strategic target of health care system reform, thus improving the current situation of fundamentally divided medical and health care systems [27]. 

The results for the Outcomes domain are reflected in the intervention coverage rate and the incidence of infectious disease. The indicators of public health intervention revealed that the reform objectives had been achieved by 2011. The data and the evaluation results indicate that tuberculosis and AIDS are still the focus of public health prevention and control. 

This study evaluated the impact of health care system reform according to residents’ health status, Economic risk-sharing and social satisfaction. Three mortality rates (Infant mortality rate, Child mortality (under 5) rate, Maternal mortality rate) in Hubei Province have declined and life expectancy has increased since the reform began. Due to the strengthening of health insurance, residents’ financial risks have decreased to a large extent. However, the participation rate for basic medical insurance among urban workers and non-working urban residents is expected to continue to improve.

## 5. Conclusions

In order to evaluate the health care system reform in Hubei Province, our study established a set of indicators including four grade-1 indicators, 16 grade-2 indicators and 76 grade-3 indicators. The effects of the reforms increased year by year from 2009 to 2011 in Hubei Province. The health status of urban and rural populations and the accessibility, equity and quality of health services in Hubei Province were improved after the reforms. 

## 6. Research Prospects

Our research has several limitations that need to be considered when interpreting the findings and that could be addressed in the future. Our results should be considered an example of what could and should be done if more data were available.

First, the indicator system needs to be adjusted in the future. The new health care system reform began in 2009. Although many studies in the literature were examined when establishing the indicator system and screening indicators, the indicator system is still imperfect because the reform was still new, data collection was limited, and some data (e.g., evaluation of the public hospital reform performance need to be set up special studies) could only evaluated qualitatively rather quantitatively. With further development of the reform and the accumulation of experience, the indicator system will need to be continuously adjusted. In future health care system evaluation studies, the weights of the indicators could be determined using other objective weighting methods so that the entire indicator system can reflect reform performance more scientifically and effectively, based on actual conditions and existing data.

Furthermore, in terms of improving data collection, as the evaluation data are derived from different projects, such as the Direct Reporting Network System, National Health Account Project, Human Resources and Social Security Department, it will be important to emphasize cooperation between different departments, use the same standards, and strengthen the system of health information for improving work efficiency and ensuring data quality.

In the end, health care system reform evaluation is significant in that it is a means rather than an end. The ultimate aim of evaluation is to uncover the achievements in and problems of health care system reform, constantly summarize experiences and address disadvantages, so that efforts can be made to promote health care system reform and development of the health system. Therefore, health care system reform evaluation should be a long-term and sustainable process with the goal of offering guidance for health care system reform and health policy in Hubei Province and in China as a whole.
